# Interstitial Pregnancy Managed with Single-dose Systemic Methotrexate: A Case Report

**DOI:** 10.31729/jnma.6595

**Published:** 2021-09-30

**Authors:** Tulasa Basnet, Punita Yadav, Manoj Kumar Sah, Jyotsna Yadav

**Affiliations:** 1 Department of Obstetrics and Gynecology, B.P. Koirala Institute of Health Sciences, Dharan, Nepal

**Keywords:** *conservative management*, *interstitial pregnancy*, *methotrexate*

## Abstract

Interstitial pregnancy is a rare type of ectopic pregnancy with a high risk of massive hemorrhage on rupture as compared to ectopic on other sites. The mortality rate for the ruptured interstitial pregnancy is high. Therefore, early diagnosis of such pregnancy before the rupture occurs facilitates appropriate management and avoids life-threatening complications. With the advancement in diagnostic imaging modalities, early diagnosis and more conservative management for interstitial pregnancy have become possible. Here, we report a case of primigravida diagnosed with interstitial pregnancy with ultrasonography and successfully managed with a single dose of methotrexate.

## INTRODUCTION

Implantation of a blastocyst into the interstitial portion of the fallopian tube results in an interstitial pregnancy. It is a rare phenomenon accounting for 2-4% of all ectopic pregnancies.^1,2^ As the uterine cornu has an abundant blood supply, it can accommodate pregnancy till later gestation. Therefore, among all the ectopic pregnancies, interstitial pregnancy is associated with the highest risk of massive hemorrhage if rupture occurs with a mortality rate of 2-2.5%.^3^ There exists a challenge differentiating interstitial pregnancy from eccentrically implanted intrauterine pregnancy.^4^ Therefore, it is imperative that the radiologist and the treating doctor be aware of the diagnostic criteria of this rare condition for timely diagnosis to avoid life-threatening complications and mortality. We report a case of interstitial pregnancy diagnosed timely with ultrasonography in a 20-year-old primigravida successfully managed with a single dose of systemic methotrexate.

## CASE REPORT

A 20-year-old primigravida presented to the emergency room of the Department of Obstetrics and Gynecology, B.P. Koirala Institute of Health Sciences at 7^1/7^ weeks of gestation with complaints of per vaginal spotting for the last three days. She did not have any other complaints at the presentation. Her vital parameters and per abdominal examination findings were normal. There was no active bleeding on per speculum examination and on bimanual examination, uterus was 6-8 weeks size, fornices were free and non-tender. The transvaginal sonography (TVS) showed a gestational sac of 7^1/7^ weeks without a fetal pole or cardiac activity, eccentrically placed in uterine fundus towards the right side surrounded by thin myometrium; suggestive of interstitial pregnancy. Her human Chorionic Gonadotropin (hCG) value at admission was 4479mIU/ml.

**Figure 1:**
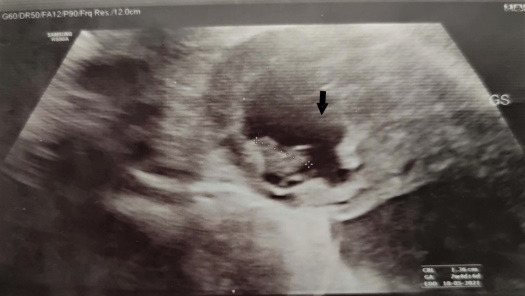
TVS on 14th day showing eccentrically placed irregular gestation sac (black arrow).

She was admitted to the ward and after explaining treatment modalities and potential risks, the patient desired to preserve the fallopian tube. Thus, medical management was planned and she received a single dose of methotrexate 62mg (50mg/m^2^). For monitoring, she was kept in the ward and β hCG was repeated on day 4 and day 7 which was 3679 and 2244 respectively. She did not develop any symptoms while she was admitted to the ward and the β hCG values were in decreasing trend. Also, her residence was nearby the hospital and she was compliant to follow up.Thus, she was discharged with advice to follow up with the β hCG report on day 11. She was also advised to report to the emergency room if she experiences any symptoms suggestive of rupture. The β hCG value on day 11 was 1111 mIU/ml. The ultrasonography (USG) was repeated after two weeks which showed irregular gestational sac-like structure placed eccentrically in the fundus without cardiac activity, suggestive of interstitial pregnancy with embryonic demise (Figure 1).

**Figure 2:**
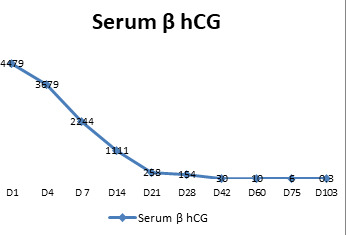
Serum β hCG value during the course of treatment and follow up.

**Figure 3:**
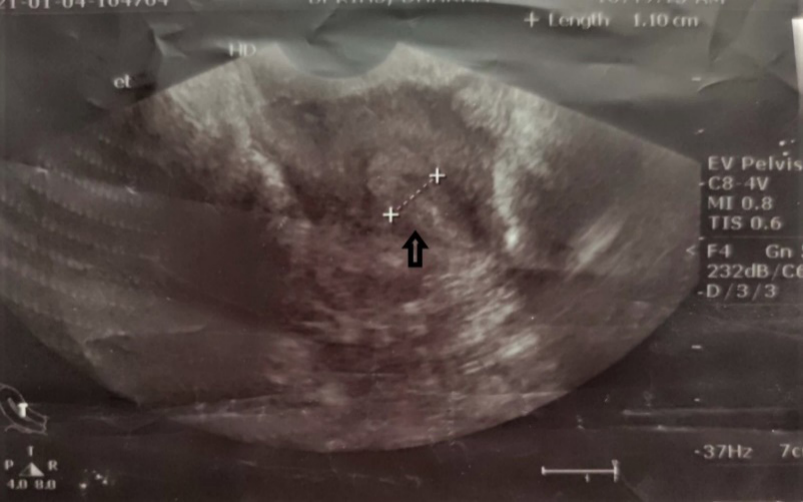
TVS on week 22 showing normal endometrial thickness (arrow).

The TVS was repeated after two weeks of discharge which showed the same finding. It was then repeated monthly. The serum β hCG was monitored two weekly and showed a decreasing pattern and became normal on day 60 (Figure 2). The TVS showed normal findings after 22 weeks of methotrexate (Figure 3). In between, she resumed her menstrual cycle after two months and had regular cycles.

## DISCUSSION

The terms "interstitial pregnancy" and "cornual pregnancy" are often used interchangeably. However, the term 'interstitial pregnancy' should only be used for the pregnancy that gets implanted into the interstitial portion of the fallopian tube and 'cornual pregnancy' should be reserved for the pregnancy in women with a single uterine horn, a bicornuate uterus, or a septate uterus.^4^

Interstitial pregnancy is suspected when eccentric implantation of the gestational sac at the superior fundal level of the uterus is detected in ultrasonography. However, there is always a difficulty in distinguishing interstitial pregnancy from eccentrically placed intrauterine pregnancy. Three diagnostic criteria of interstitial pregnancy in sonography are: (a) Empty uterine cavity; (b) Gestational sac separated <1 cm from the most lateral edge of the uterine cavity, and (c) Thin myometrial layer surrounding the gestational sac.^4^ The ultrasonography has reported sensitivity of 80% and specificity of 99% for the diagnosis of interstitial pregnancy.^2^ If the finding of USG is inconclusive, MRI can be of assistance. Another modality for diagnosis is laparoscopy, which has the advantage of allowing treatment at the same time as well.^5^

Different surgical and non-surgical treatment modalities are available for the management of interstitial pregnancy. However, the choice of management depends on the patient's symptoms, hemodynamic stability, time of diagnosis, size of pregnancy, clinical expertise as well as the desire for fertility preservation.^3^ If the interstitial pregnancy ruptures, laparotomy for the repair of defect or hysterectomy, in difficult cases, is often needed. Successful laparoscopic management for ruptured interstitial pregnancies is also being reported increasingly. Nevertheless, the route of surgery depends on the surgical expertise and availability of the facilities.

The facilities for assessment of pregnancy at an earlier gestation, high-resolution transvaginal sonography, availability of quantitative β hCG level have enabled us to detect ectopic pregnancy before rupture occurs and manage them conservatively. Our patient also presented to us in the seventh week. Because of early presentation and diagnosis, conservative management was a feasible option for our patient. Conservative management may again be surgical or medical. Conservative surgical management includes laparoscopic resection of the pregnancy and involved tube with preservation of the uterine architecture.^6^

Conservative medical management uses methotrexate through various routes like systemic, injection into the sac under ultrasound guidance through transvaginal or transabdominal route, hysteroscopic injection into the sac, or combined local and systemic route.^7^ Dilatation and curettage under ultrasound guidance followed by a single dose of systemic methotrexate is also reported as a fertility-preserving method of management.^8^ However, conservative management should only be offered to those patients with radiological diagnosis of interstitial pregnancy, who do not have risk factors for immediate rupture and are hemodynamically stable. Also, close ultrasonographic and β hCG follow-up and good clinical acumen are must for conservative management.^3^ As our patient fulfilled all these criteria and was compliant to follow up, she proved to be an ideal candidate for conservative management. Also, there is always some risk of uterine rupture at the site of cornual repair in subsequent pregnancy.^9^

Following Methotrexate treatment serum β hCG is reported to normalize by two months but gestational sac may be visible in USG for up to 4 months.^7^ In our patient, β hCG normalized after eight weeks and gestational sac in TVS disappeared at 22 weeks of treatment. During the course of treatment, she did not develop any complications, resumed menses after two months of treatment, and had regular cycles. However, she always had a concern regarding the persistence of the gestational sac for a long duration even after normalization of β hCG. We had counseled her at the start of treatment that resolution of the gestational sac may even take up to six months based on the literature. It took five months for the sac to disappear in her case. She was then advised that she can plan her next pregnancy after 6 months and visit for an early pregnancy checkup once she conceives again.

Early detection of interstitial pregnancy allows for conservative management with Methotrexate in the hemodynamically stable patient if proper follow-up with β hCG and TVS can be done and emergency surgical management is available in case the need arises. Conservative management not only prevents the morbidity associated with anesthesia and surgical procedure but also avoids uterine scar and risk of rupture of the scar in the subsequent pregnancy. Having said so, the prerequisites and facility for proper follow-up should be ensured before deciding for conservative management.
